# Pharmacotherapy of motor symptoms in early and mid-stage Parkinson’s disease: guideline “Parkinson’s disease” of the German Society of Neurology

**DOI:** 10.1007/s00415-024-12632-6

**Published:** 2024-08-29

**Authors:** Matthias Höllerhage, Jos Becktepe, Joseph Classen, Günther Deuschl, Georg Ebersbach, Franziska Hopfner, Paul Lingor, Matthias Löhle, Sylvia Maaß, Monika Pötter-Nerger, Per Odin, Dirk Woitalla, German Parkinson’s Guidelines Group, Matthias Höllerhage, Jos Becktepe, Günther Deuschl, Georg Ebersbach, Franziska Hopfner, Paul Lingor, Matthias Löhle, Sylvia Maaß, Monika Pötter-Nerger, Per Odin, Dirk Woitalla, Mathias Bähr, Daniela Berg, Kathrin Brockmann, Carsten Buhmann, Andrés Ceballos-Baumann, Joseph Claßen, Cornelius Deuschl, Richard Dodel, Carsten Eggers, Thilo van Eimeren, Alessandra Fanciulli, Bruno Fimm, Ann-Kristin Folkerts, Madeleine Gausepohl, Alkomiet Hasan, Wiebke Hermann, Rüdiger Hilker-Roggendorf, Günter Höglinger, Wolfgang Jost, Elke Kalbe, Jan Kassubek, Stephan Klebe, Christine Klein, Martin Klietz, Thomas Köglsperger, Andrea Kühn, Paul Krack, Florian Krismer, Gregor Kuhlenbäumer, Johannes Levin, Inga Liepelt-Scarfone, Kai Loewenbrück, Stefan Lorenzl, Walter Maetzler, Regina Menzel, Philipp T. Meyer, Brit Mollenhauer, Manuela Neumann, Tiago Outeiro, René Reese, Kathrin Reetz, Olaf Rieß, Viktoria Ruf, Anja Schneider, Christoph Schrader, Alfons Schnitzler, Klaus Seppi, Friederike Sixel-Döring, Alexander Storch, Lars Tönges, Thilo van Eimeren, Uwe Walter, Tobias Wächter, Tobias Warnecke, Florian Wegner, Christian Winkler, Karsten Witt, Kirsten Zeuner, Claudia Trenkwalder, Günter U. Höglinger

**Affiliations:** 1 Department of Neurology, Hannover Medical School, Hannover, Germany; 2 Department of Neurology, Christian-Albrechts-University, Kiel, Germany; 3 Department of Neurology, Leipzig University Medical Center, Leipzig, Germany; 4 Movement Disorders Hospital, Beelitz-Heilstätten, Germany; 5 Department of Neurology with Friedrich Baur Institute, LMU University Hospital, Ludwig-Maximilians-Universität (LMU) München, Marchioninistr. 15, 81377 Munich, Germany; 6 School of Medicine and Health, Department of Neurology, Technical University of Munich, Klinikum rechts der Isar, Munich, Germany; 7 Munich Cluster for Systems Neurology (SyNergy), Munich, Germany; 8 German Center for Neurodegenerative Diseases (DZNE), Munich, Germany; 9 Department of Neurology, University of Rostock, 18051 Rostock, Germany; 10 Deutsches Zentrum für Neurodegenerative Erkrankungen (DZNE) Rostock/Greifswald, Rostock, Germany; 11 Department of Neurology, Universitätsklinikum Hamburg-Eppendorf, Hamburg, Germany; 12 Division of Neurology, Lund University, Lund, Sweden; 13 Department of Neurology, Skåne University Hospital, Lund, Sweden; 14 Department of Neurology, St. Josef-Hospital, Katholische Kliniken Ruhrhalbinsel, Contilia Gruppe, Essen, Germany; 15 Paracelsus-Elena-Klinik, Kassel, Germany; 16 Department of Neurosurgery, University Medical Center, Göttingen, Germany

**Keywords:** Parkinson's disease, Pharmacotherapy, Guidelines

## Abstract

**Background and objective:**

There are multiple pharmacological treatment options for motor symptoms of Parkinson’s disease (PD). These comprise multiple drug classes which are approved for the condition, including levodopa, dopamine agonists, COMT inhibitors, MAO-B inhibitors, NMDA-receptor antagonists, anticholinergics, and others. Some of the drugs are approved for monotherapy and combination therapy while others are only approved as adjunctive therapy to levodopa. Furthermore, treatment for special treatment situations, e.g., rescue medication for off-phases, for tremor, treatment during pregnancy and breast feeding is discussed and recommendations are given with further details.

**Methods:**

The recommendations were based on systematic literature reviews, drafted by expert teams, consented in online polls followed by online consensus meetings of the whole German Parkinson’s Guideline Group, and publicly released in November 2023.

**Results:**

In the new S2k (i.e., consensus-based) guidelines, the pharmacotherapy of the motor symptoms of PD is discussed in five chapters. These comprise “Parkinson medication”, “Initial monotherapy”, “Early combination therapy”, “Fluctuations and dyskinesia”, and “Parkinsonian tremor”. Furthermore, there is a chapter for special treatment situations, including perioperative management, freezing of gait, and pregnancy and breastfeeding.

**Conclusion:**

The recommendations for the pharmacotherapy of motor symptoms of PD have been updated. Newly available drugs have been added, while other drugs (e.g., ergoline dopamine agonists, anticholinergics, budipine) have been removed from the recommendations.

## Introduction

There are multiple drugs and drug classes that are approved for the treatment of motor symptoms of Parkinson’s disease (PD). These comprise drugs used to substitute the dopamine deficit (e.g., levodopa, dopamine agonists), compounds that inhibit dopamine degradation (e.g., MAO-B inhibitors, COMT inhibitors), as well as NMDA-receptor antagonists, and anticholinergic compounds. Some drugs are approved as monotherapy, while others are only approved as adjunctive therapy to levodopa. The guideline is an abbreviated translation of the German guidelines “Parkinson’s disease” of the German Society of Neurology. This article includes all chapters and recommendations from the guidelines that address the pharmacotherapy of motor symptoms in PD. In the German guidelines, pharmacotherapy of motor symptoms is addressed in five chapters: “Parkinson Medication,” “Initial Monotherapy,” “Early Combination Therapy,” “Fluctuations and Dyskinesia,” and “Parkinsonian Tremor.” Furthermore, there is a chapter for special treatment situations, including perioperative management, freezing of gait, and pregnancy and breastfeeding (part VI of this article). Device-assisted therapies, such as pumps and deep brain stimulation, are covered in a different article in this article collection.

In the new guidelines, all recommendations have been reviewed and, where necessary, were updated or newly written. Drugs that have been approved since the last version of the guidelines have now been included in the recommendations (e.g., opicapone, safinamide), while others were considered obsolete or with unacceptable side effects and have therefore been removed from the recommendations (e.g., ergoline dopamine agonists, anticholinergics, budipine). The guidelines include tables that summarize studies, reviews or meta-analysis conducted with dopamine agonist and MAO-B-inhibitors. Furthermore, the new guidelines comprise an updated version of an equivalent dose table, including newly approved drugs.

## Methodology

The new German guidelines “Parkinson’s disease” are S2k guidelines, in which “k” stands for the German word “Konsensus”, meaning that they are based on a consensus by a group of experts. “S2” means that the guidelines have been developed by a structured process to find this consensus which is described briefly as follows. Primarily, key PICO (patient, intervention, comparison, outcome) [1] questions for the chapters were defined by the steering committee and modified by the author groups of the individual chapters. According to these, a systematic literature search was performed, identifying relevant studies, reviews, and meta-analysis. The literature found has been supplemented with sources identified by the authors of the individual chapters. Background texts and recommendations were written by the author groups of the individual chapters and an online vote of all members of the German Parkinson Guideline Group was conducted. Recommendations with less than 85% consensus were discussed in online consensus meetings of the German Parkinson Guideline Group. Approval of > 95% was considered a “strong consensus” and between 75 and 95% a “consensus”. Recommendations in this article are expressed as “should” (strong recommendation), “can” (less strong recommendation), or “may be considered” (even less well-established recommendation).

The full guideline was released in November 2023 by the DGN (www.dgn.org) and the Arbeitsgemeinschaft wissenschaftlicher Medizinischer Gesellschaften (AWMF, https://register.awmf.org/de/leitlinien/detail/030-010). This article presents an abbreviated and translated version of the chapters of the guideline dealing with the pharmacotherapy of the motor symptoms in early and mid-stages of PD.

## Recommendations

### Part I—which drugs are available for motor symptoms of PD?

This part of the article covers the recommendations for the drugs that are available for the treatment of motor symptoms of PD. The recommendations for the use of these drugs in different treatment situations are covered in the second part of this article.

#### Which properties influence the prioritization of specific levodopa preparations for individual patients?

*Background: *There are different pharmacokinetic formulations of levodopa available, i.e., oral standard-release, rapidly dissolving or extended-release formulations and an inhalable formulation. The oral formulations, but not the inhalable formulation, are always combined with a dopa decarboxylase inhibitor, either carbidopa or benserazide.

*Results:* There is no published evidence that extended-release formulations of levodopa with dopa decarboxylase inhibitors are superior to the standard-release formulation in the treatment of PD. There are no studies that compared 4:1 ratio combinations of levodopa/carbidopa with 4:1 ratio levodopa/benserazide. In a study from 1999, standard (immediate release) was compared with extended-release levodopa/carbidopa, and no difference in symptom control, development of fluctuations, or dyskinesias was found within the 5-year observation period [2]. Due to the lack of comparative studies, no recommendation can be made regarding a preference for the decarboxylase inhibitor. Extended-release preparations do not offer any advantage over standard-release preparations in terms of symptom control, motor fluctuations, or dyskinesias.

**Table:** 

Recommendation (new in German guideline, 2023)
Levodopa preparations can be used for the treatment of PD with regard to the specific indications
With regard to published data, there is no prioritization of preparations with the dopa decarboxylase inhibitor carbidopa or benserazide
Extended-release formulations of levodopa with dopa decarboxylase inhibitors should not be used to treat patients during waking hours, but only to alleviate symptoms during nighttime
Rapidly dissolving oral and inhalable levodopa formulations can be used to manage off-periods; however, inhalable levodopa can only be used in combination with an oral levodopa preparation as it is not combined with a dopa decarboxylase inhibitor
Level of consensus: 92.9%, consensus

#### What influences the prioritization of different dopamine agonists for individual patients?

*Background:* Non-ergoline dopamine agonists (such as pramipexole, ropinirole, piribedil, rotigotine, and with significant limitations apomorphine) are generally approved as first-line therapy. Pramipexole and ropinirole are also available as extended-release tablets. Rotigotine is available as transdermal patch. Apomorphine is available as pens or pumps for subcutaneous injections or infusions or as sublingual films.

*Results*: There are no recent controlled studies comparing the effectiveness or side effects of individual dopamine agonists and there is no evidence for differences in efficacy of the four non-ergoline dopamine agonists. There is no evidence that ergoline dopamine agonists are more effective when other approved substances or non-ergoline dopamine agonists do not provide satisfactory symptom control. Therefore, the use of ergoline dopamine agonist is not recommended anymore due to their side effect profile. In a double-dummy study comparing pramipexole and rotigotine, skin reactions occurred more frequently with rotigotine, while no difference of efficacy was detected [3]. Piribedil is metabolized in the liver and mainly excreted via the kidneys. Rotigotine is mainly metabolized by various CYP isoenzymes and is then excreted via the kidneys. Pramipexole is mainly excreted via the kidneys. Dose adjustment is recommended for patients with impaired kidney function. Ropinirole is metabolized via the Cytochrome P450 isoenzyme CYP1A2. Dose adjustment is not required for moderate kidney function impairment.

**Table:** 

Recommendation (new in German guideline, 2023)
Ergoline dopamine agonists (bromocriptine, cabergoline, pergolide) should no longer be used for the treatment of PD
Non-ergoline dopamine agonists (pramipexole, ropinirole, piribedil, rotigotine, with limitations apomorphine) can be used for the treatment of PD, taking into account the specific indications outlined below
Apomorphine is available for subcutaneous injection or infusion, or as a sublingually applicable film, and is therefore tied to specific indications
Pramipexole and ropinirole in extended-release tablet formulations, and rotigotine as a transdermal patch, allow for once-daily dosing
Prioritizing different dopamine agonists in terms of effectiveness cannot be definitively derived from the literature
When concomitantly medicated with drugs that induce or inhibit CYP1A2, dose adjustment of ropinirole or switching to another dopamine agonist should be considered
In cases of impaired liver function, prioritizing pramipexole, which is primarily metabolized via the kidneys, should be considered
In cases of impaired kidney function, prioritizing ropinirole, rotigotine, or piribedil over pramipexole should be considered
Level of consensus: 96%, strong consensus

#### What influences the prioritization of individual COMT inhibitors for individual patients?

*Background*: Three COMT inhibitors (entacapone, tolcapone, and opicapone) are available for the treatment of PD [4, 5]. Entacapone can be administered up to ten times daily with each levodopa intake. Tolcapone is given up to three times daily. Opicapone is administered once daily in the evening [6, 7].

*Results*: There is no direct comparative study of the COMT inhibitors against each other. However, there is a meta-analysis [4] comparing the efficacy of the three COMT inhibitors and a recent review article [5]. In the recent meta-analysis, entacapone, opicapone, and tolcapone all significantly extended on-time and increased dyskinesia compared to placebo. Tolcapone showed the best efficacy in extending on-time, while opicapone showed a longer extension of on-time than entacapone, although not statistically significant. The side effect rate was highest with tolcapone, followed by entacapone and then opicapone. All COMT inhibitors are effective in reducing off-time [4].

**Table:** 

Recommendation (new in German guideline, 2023)
Opicapone and entacapone, as COMT inhibitors, are largely equivalent in their effects and can be used for the treatment of fluctuations in PD
Tolcapone should only be used as a second-line agent due to hepatotoxicity and should be closely monitored for safety (clinically and with laboratory tests)
Level of consensus: 100%, strong consensus

#### What influences the prioritization of individual MAO-B-inhibitors for individual patients?

*Background*: The MAO-B-inhibitors rasagiline and selegiline are established in the monotherapy of PD and in combination with levodopa for motor fluctuations. Safinamide has a dual mechanism of action, inhibiting MAO-B at both approved dosages (50 mg and 100 mg) and additionally acting glutamatergically at the higher dosage. Safinamide is approved only in combination with levodopa for treatment of motor fluctuations.

*Results*: There are no studies, in which the MAO-B inhibitors rasagiline and selegiline have been directly compared with each other. Safinamide is not approved for monotherapy.

**Table:** 

Recommendation (new in German guideline, 2023)
It is not possible to prioritize the different MAO-B inhibitors in terms of their efficacy based on the literature
The MAO-B-inhibitors selegiline or rasagiline can be used as monotherapy for early PD or in combination with levodopa for treating PD with motor fluctuations
Safinamide, a MAO-B-inhibitor with a dual mechanism of action, is not approved as a monotherapy but can be used in combination with levodopa for treating PD with motor fluctuations
Level of consensus: 100%, strong consensus

#### What influences the prioritization of individual NMDA-receptor antagonists for individual patients?

*Background*: The NMDA-receptor receptor antagonist amantadine is available for the therapy of PD. Budipine is no longer marketed in Germany.

*Results*: There is a systematic analysis from 2003 [8] and a recent review [9] on amantadine in the therapy of PD. Amantadine has a positive effect on levodopa-induced dyskinesia. There is insufficient evidence for the efficacy and safety of amantadine in the early phase of PD [8, 9].

**Table:** 

Recommendation (new in German guideline, 2023)
Amantadine can be used for the treatment of PD, considering the specific indications outlined below
Budipine is no longer recommended due to its side effect profile
Level of consensus: 95.2%, strong consensus

#### What influences the prioritization of individual anticholinergics for individual patients?

*Background*: Anticholinergics (biperiden, bornaprine, metixene, trihexyphenidyl) are approved medications for the therapy of PD.

*Results*: There are only insufficient data from randomized studies on the efficacy and tolerability of anticholinergics in PD and no recent RCTs or meta-analyses are available. The effectiveness of anticholinergics appears to be rather weak.

**Table:** 

Recommendation (new in German guideline, 2023)
Anticholinergics should not be used as anti-Parkinson's agents due to an unfavorable benefit-risk profile compared to alternative therapies
In very exceptional cases, their use may be considered for tremor
Level of consensus: 96.4%, strong consensus

#### What dosage guidelines apply to dopamine agonists and MAO-B-inhibitors?

*Background*: The various medications for the motor symptoms of PD are available in different dosages.

*Results*: The dosage recommendations according to the European approvals for non-ergot-derived-dopamine agonists are presented in Table 1.

**Table 1:** Dosage recommendation for non-ergoline dopamine agonists

Drug	Starting regimen	Weekly increase in dosage	Maintenance dose	Total and maximal daily dose
Piribedil	50 mg in the evening	50 mg every 2 weeks	2 to 3 × 50 mg, until 100–50–100 mg	150 mg to 250 mg
Pramipexole standard-release	3 × 0.088 mg	2nd week: 3 × 0.18 mg 3rd week: 3 × 0.35 mg Weekly increase by 3 × 0.18 mg	3 × 0.35 mg to 3 × 0.7 mg	1.05 mg to 3.3 mg
Pramipexole extended-release	0.26 mg in the morning	2nd week: 1 × 0.52 mg 3rd week: 1 × 1.05 mg, weekly increase up to 1 × 2.1 mg or 1 × 3.15 mg	1 × 1.05 mg to 1 × 2.1 mg	1.05 mg to 3.15 mg
Ropinirole standard-release	1 mg in the morning	1 mg; from 6 mg daily dose on, weekly increase by 1.5 to 3 mg	3 × 3 mg to 3 × 8 mg	6 mg to 24 mg
Ropinirole extended-release	2 mg in the morning	2 mg	1 × 6 mg to 24 mg	6 mg to 24 mg

The MAO-B-inhibitor rasagiline is approved at 1 mg once daily. Selegiline is available as tablets in doses of 5 mg or 10 mg. The half-life spans several days, and a dose–response relationship cannot be derived from the available studies. For the dosage of safinamide, the data for 200 mg are very limited. Doses between 50 and 100 mg are recommended as add-on therapy to levodopa, and a dose-dependent difference in efficacy could not be demonstrated [10, 11]. The dosage recommendations for the MAO-B-inhibitors are presented in Table 2.

**Table 2:** Dosage recommendation for MAO-B-inhibitors

Drug	Starting regimen	Weekly increase in dosage	Maintenance dose	Total daily dose
Rasagiline	1 × 1 mg	None	1 × 1 mg	1 mg
Selegiline	1 × 5 mg	5 mg	1 to 2 × 5 mg/1 × 10 mg	5 to 10 mg
Safinamide	1 × 50 mg	For off-symptoms: 50 mg For dyskinesia: 100 mg	1 × 50 to 100 mg	50 to 100 mg

The dosing recommendations apply in the outpatient setting for treating healthcare providers who are not specifically specialized in movement disorders. In in-patient-settings or in specialized movement disorders centers, faster titration may be considered.

#### What is the levodopa equivalent dose of each Parkinson's medication (dopamine agonists, MAO-B-inhibitors, COMT inhibitors, amantadine, anticholinergics)?

*Background*: The available medications for the symptoms of PD differ significantly in their dosage ranges, prompting the question of equivalent doses.

*Results*: There are three publications with equivalent dose tables [12–14]. A summary is shown in Table 3.

**Table 3:** The table includes the drug classes, the drugs, the equivalent dose to 100 mg of levodopa, and, for simplicity, the multiplier by which the drug dose must be multiplied to calculate the equivalent dose

Drug class	Drug	Single doses (mg/100 mg levodopa)	Multiplier
Levodopa	Levodopa (LD)	100	1
	Extended-release levodopa	133	0.75
	Levodopa-carbidopa intestinal gel (LCIG)	90	1.11 (morning dose and maintenance dose)
	Levodopa-entacapone-carbidopa intestinal gel (LECIG)	90	1.11 (morning dose)
		70	1.46 (maintenance dose)
COMT inhibitors*	Entacapone	LD × 0.33	LD × 0.33
	Tolcapone	LD × 0.5	LD × 0.5
	Opicapone	LD × 0.5	LD × 0.5
Dopamine agonists (non-ergoline)	Pramipexole	1 mg salt	100
	Ropinirole	5	20
	Rotigotine	3.3	30
	Piribedil	100	1
	Apomorphine (infusion or injection)	10	10
MAO-B-inhibitors	Selegiline 10 mg (orally)	10	10
	Selegiline 1.25 mg (sublingual)	1.25	80
	Rasagiline	1	100
	Safinamide	100–150	0.66–1
Others	Amantadine	100	1

*To calculate the equivalent dose of COMT inhibitors, the total levodopa dose (including extended-release levodopa) is multiplied by the corresponding value and added to the total dose of levodopa

### Part II—initial monotherapy

#### When is pharmacotherapy for Parkinson’s disease indicated?

*Background*: The question of when to start pharmacotherapy in individuals with Parkinson's disease depends on a variety of therapeutic goals.

*Results*: There are no systematic studies that have investigated when the indication for pharmacotherapy of PD is given. The recommendations from the previous German guideline were based on expert opinion and were not changed in the new guidelines.

**Table:** 

Recommendation (in German guideline, 2023)
Pharmacotherapy for PD should begin timely, tailored to the patient's age, and efficiently. Depending on age, duration of illness, and social situation, the following therapeutic goals may become relevant:
1. Treatment of motor and/or non-motor and/or autonomic disturbances
2. Management of behavioral and psychological symptoms of the disease
3. Preservation of independence in activities of daily living
4. Prevention/reduction of dependency on care
5. Preservation of independence within family and society (social competence)
6. Maintenance of employability
7. Preservation/increase of quality of life
8. Prevention of secondary orthopedic and internal medical conditions
9. Prevention/treatment of motor and non-motor complications
10. Avoidance of dopaminergic side effects
Level of consensus: 100%, strong consensus

#### How effective and safe is standard-release levodopa compared to placebo in the monotherapy of early-stage PD?

*Background*: While the standard therapy for PD comprises levodopa, the use of levodopa preparations is associated with motor complications in more advanced stages of PD, particularly levodopa-induced dyskinesia or fluctuations in therapeutic effectiveness, also known as “wearing-off” or “end of dose” hypokinesia. These fluctuations are initially predictable, but may become unpredictable in the course of the disease. Delaying levodopa use in younger patients is discussed to prevent complications, but dyskinesia development is mainly influenced by high total daily doses, disease duration, and the degree of the degenerative process. Eventually, most PD patients require levodopa for symptom control, with approximately 50–90% needing it within 4–6 years of diagnosis. Long-term use becomes essential for symptom management in the majority of patients [15, 16].

*Results*: The main basis for the effectiveness of levodopa plus dopa decarboxylase inhibitor has been provided by the ELLDOPA study. In this study, levodopa reduced symptoms of PD in a dose-dependent manner [17]. A number of studies have suggested that levodopa therapy should be delayed due to the development of motor fluctuations [16]. In 2014, a study systematically examined the relationship between disease duration, levodopa dose, duration of therapy, age, and the development of motor fluctuations and dyskinesias, comparing patients from Ghana and Italy. Regarding the development of motor fluctuations and dyskinesias, higher daily levodopa dose and total disease duration were factors contributing to the development of motor fluctuations and dyskinesias, while the disease duration at the start of levodopa therapy had no influence [15]. A meta-analysis examining 14 RCTs could not prove a linear relationship between the levodopa dose. However, the authors state that the existence of levodopa-induced dyskinesia is confirmed by everyday clinical experience [18]. A new randomized double-blind study confirmed that there is no evidence that levodopa has an impact on disease progression of PD [19]. In summary, levodopa is still considered to be the most effective and a safe treatment for PD. Short-term dopaminergic side effects are rare and usually temporary. However, long-term therapy with levodopa can dose-dependently contribute to motor complications, such as fluctuations and dyskinesias.

**Table:** 

Recommendation (new in German guideline, 2023)
Levodopa can be used for monotherapy in early-stage PD, considering the specific differential indications listed below
Levodopa should be administered at the lowest effective dose possible
Based on the available evidence, levodopa has neither a negative nor a positive effect on disease progression
Level of consensus: 91%, consensus

#### How effective and safe are dopamine agonists compared to placebo or levodopa in the monotherapy of early-stage PD?

*Background*: Dopamine agonists are also available for the monotherapy of PD.

*Results*: There are numerous trials, reviews and meta-analyses available. Since the publication of the last guidelines, a review [20] and a meta-analysis [21] on impulse control disorders under dopaminergic agonists have been published. The characteristics and results of the major trials conducted with dopamine agonists are summarized in Table 4. The characteristics and results of reviews and meta-analyses are summarized in Table 5. The trials, reviews, and meta-analyses consistently demonstrate that dopamine agonists are effective compared to placebo in the treatment of early-stage PD. Regarding the comparison with levodopa, the data basis is sparse.

**Table 4:** Summary of randomized controlled trials on the effectiveness of dopamine agonists in the early stage of PD

Publication	Number of patients (*n*)	Dopamine agonist vs. placebo or levodopa	Duration of follow-up	Efficacy vs. placebo	Efficacy vs. levodopa	Safety: fluctuations	Compatibility: dyskinesia	Compatibility: non-motor adverse drug reaction	Methodological weaknesses/comments
Zesiewicz et al. 2017 [22]	Placebo: *n* = 40, ropinirole: 2 mg: *n* = 13, 4 mg: *n* = 41, 8 mg: *n* = 40, 12 mg, *n* = 39, 24 mg: *n* = 13	Ropinirole vs. placebo	Titration for 13 to 17 weeks, followed by 4 to 7 weeks of maintenance dose, 1 week of dose reduction, 1 to 2 weeks of wash out	Ropinirole superior, only significant in one dosage group	Not investigated	Not reported	One patient with ropinirole, no patient with placebo	Nausea, sleepiness, headaches, dizziness, hypertension, vomiting	Many subgroups leading to small patient numbers in each group, short study duration
Hauser et al. 2016b [23]	Placebo: *n* = 40, rotigotine (≤ 6 mg): *n* = 41 Rotigotine (≥ 8 mg): *n* = 41	Rotigotine vs. placebo	Titration for 4 to 7 weeks, followed by 12 weeks of maintenance dose, up to 12 days of dose reduction, 4 weeks of wash out	Rotigotine superior	Not investigated	Not reported	Not more frequently compared to placebo	Not more frequently compared to placebo	Short study duration
Rascol et al. 2006b [24]	Placebo: *n* = 204 Piribedil: *n* = 197	Placebo vs. piribedil	24 months	Piribedil superior		Not significant		Nausea, hypotension, sleepiness	Surprisingly strong effect of piribedil, many centers in India, Mexico, and Argentina
Rascol et al. 2006a [25]	Ropinirole: *n* = 179 Levodopa: *n* = 89	Ropinirole vs. levodopa	5 years			Not reported	Ropinirole superior	Not reported	Follow-up study with small number of completers
Watts et al. 2007 [26]	Rotigotine: *n* = 181 Placebo: *n* = 96	Rotigotine vs. placebo	27 weeks	Rotigotine superior				Local skin reactiong, dizziness, nausea, headaches	(Last observation carried forward) LOCF instead of ITT, sex differences without explanation
Jankovic et al. 2007 [27]	Rotigotine: *n* = 181 Placebo: *n* = 96	Rotigotine vs. placebo	27 weeks						Double publication
Poewe et al. 2011 [28]	Placebo: *n* = 103 Pramipexole extended-release: *n* = 223 Pramipexole immediate-release: *n* = 213	Pramipexole extended-release vs. pramipexole standard-release vs. placebo	33 weeks including titration period	Pramipexole extended release (ER) and pramipexole immediate release (IR) superior				Sleepiness, nausea, constipation, dizziness, dryness of mouth	
Mizuno et al. 2013 [29]	Rotigotine: *n* = 82 Placebo: *n* = 90	Rotigotine vs. placebo	12 weeks including titration period	Rotigotine superior		Two deaths under rotigotine (not related to therapy)		Not reported in original publication	Drop-out-rate relatively high
Schapira et al. 2013 [30]	Placebo: *n* = 261 Pramipexole: *n* = 274	Pramipexole vs. placebo	15 months, delayed start. Verum after 9 (6) months	Not significant				Nausea, edema, sleepiness, hallucinations	Only a part of the patients received FP-CIT imaging

**Table 5:** Summary of meta-analyses and systematic reviews on the effectiveness of dopamine agonists in the early stage of PD

Publication	Number of trials	Dopamine agonists vs. placebo or levodopa	Efficacy vs. placebo	Efficacy vs. levodopa	Safety: fluctuations	Compatibility: dyskinesia, dystonia	Compatibility: non-motor adverse drug reaction	Methodological weaknesses/comments
Chen et al. 2017 [31]	12	Rotigotine vs. placebo	Rotigotine superior	Not investigated	Not reported	More frequently under rotigotine in one study	ADR more frequently under rotigotine	
Giladi et al. 2016 [32]	6	Rotigotine vs. placebo, rotigotine plus levodopa vs. placebo	Rotigotine superior (in all disease stages)	Not investigated	Not investigated	Not investigated	Not investigated	Inclusion of trials with early-stage patients (no previous levodopa therapy) and trials with advanced-stage patients (levodopa therapy as inclusion criterion)
Shen and Kong 2018 [33]	3	Pramipexole standard-release vs. extended-release vs. placebo						Small number of studies, only 2 trial with placebo group
Márquez-Cruz et al. 2016 [34]	5	Levodopa vs. placebo, pramipexole vs. placebo, rasagiline vs. placebo, selegiline vs. placebo	Pramipexole superior	Levodopa superior	Not reported	More frequently under levodopa	No significant differences	Only pramipexole as dopamine agonist
Binde et al. 2020 [35]	79	4 dopamine agonist (cabergoline, pramipexole, ropinirole, rotigotine) vs. 3 MAO-B-inhibitors (selegiline, rasagiline, safinamide (as add-on)) vs. levodopa vs. placebo	Ropinirole, pramipexole, rotigotine, cabergoline superior	Ropinirole superior Pramipexole, rotigotine, cabergoline inferior	Not reported	More frequently under levodopa	More frequently under pramipexole, no significant differences with other drugs	In the study relative efficacy values were calculated
Zhuo et al. 2017 [36]	24	Levodopa, different dopamine agonists, different MAO-B-inhibitors	Not investigated	Not investigated		More frequently under levodopa and dopamine agonists compared to placebo	Nausea, sleepiness, dizziness more frequently under dopamine agonists compared to placebo, hallucinations more frequently under ropinirole vs. placebo	Only investigation of side effects, no investigation of clinical efficacy
Kulisevsky and Pagonabarraga 2010 [37]	40	Dopamine agonists vs. placebo and vs. each other	Not investigated	Not investigated		Ropinirole superior to levodopa	Differences between dopamine agonists	Quality of included studies not considered as good
Stowe et al. 2008 [38]	21	Dopamine agonists vs. placebo or levodopa	Dopamine agonist superior	Levodopa superior	Dopamine agonist superior	Dopamine agonist superior	Levodopa superior	Very good cochrane analysis
Fox et al. 2011 [39]	68, not all related to the question	Dopamine agonists vs. placebo or levodopa	Piribedil, pramipexole, ropinirole, rotigotine, cabergoline, pergolide superior					Systematic review as basis for the treatment recommendation of the Movement Disorders Society
Baker et al. 2009 [40]	25	Dopamine agonists vs. placebo or levodopa	Dopamine agonist superior	Levodopa superior	Dopamine agonist superior	Dopamine agonist superior	Levodopa superior	Meta-analysis, appears to overestimate the effect of dopamine agonists

**Table:** 

Recommendation (new in German guideline, 2023)
Non-ergoline dopamine agonists can be used for the initial monotherapy of early PD
A non-ergoline dopamine agonist should be titrated up to a clinically effective but still well-tolerated dose
If side effects occur with a non-ergoline dopamine agonist, preventing an effective therapy, another class of substance (levodopa or MAO-B-inhibitors) or another non-ergoline dopamine agonist should be used for initial monotherapy
Level of consensus: 100%, strong consensus

#### How effective and safe are MAO-B-inhibitors compared to placebo or levodopa or dopamine agonists in the monotherapy of PD in the early stage?

*Background*: The MAO-B-inhibitors rasagiline and selegiline are established in the monotherapy of PD, while safinamide is not approved for monotherapy.

*Results*: Since 2005, no studies have been published comparing the effectiveness of MAO-B-inhibitors with levodopa in the treatment of early-stage PD. The results of RCTs investigating MAO-B-inhibitors are summarized in Table 6. Recent reviews and meta-analyses are summarized in Table 7. Recent meta-analyses and RCTs confirmed that MAO-B-inhibitors are effective in the treatment of early-stage PD. The strongest evidence is available for rasagiline. Studies directly comparing the clinical effects of MAO-B-inhibitors, dopamine agonists, and levodopa do not exist.

**Table 6:** Summary of randomized controlled trials of MAO-B-inhibitors in early-stage PD

Publication	Number of patients	Drug vs. placebo or levodopa	Duration of follow-up	Efficacy vs. placebo	Efficacy vs. levodopa	Safety: fluctuations	Compatibility: dyskinesia	Compatibility: non-motor adverse drug reaction	Methodological weaknesses/comments
Elgart et al. 2019 [41]	64 healthy persons	Not investigated		Not investigated	Not investigated	Not investigated	Not investigated	No clear difference between rasagiline and placebo	Pharmakokinetics and safety study, single center, healthy persons, comparison between 32 Japanese with 32 Caucasian persons
Hattori et al. 2019 [42]	Rasagiline: *n* = 118 Placebo: *n* = 126	Rasagiline	26 weeks	Rasagiline superior	Not investigated	Nicht berichtet	Not investigated	Headaches and eczema more frequently with rasagiline	Multi-center study, conducted in Japan
Mizuno et al. 2017 [43]	Selegiline: *n* = 146 Placebo: *n* = 146	Selegiline	12 weeks	Selegiline superior	Not investigated	Not investigated	Not investigated		Multi-center study, conducted in Japan
Biglan et al. 2006 [44]	Placebo: *n* = 132 Rasagiline: *n* = 124 (1 mg), *n* = 124 (2 mg)	Placebo vs. rasagiline, rasagiline vs. rasagiline	6 months placebo vs. rasagiline, followed by 12 months rasagiline vs. rasagiline	Rasagiline superior in both concentrations in early start					Over-estimation, no information about confounding or randomization, insufficient description of blinding process, high drop-out rate
Hauser et al. 2009 [45]	Placebo: *n* = 138 Rasagiline (1 mg): *n* = 128 (early start) and *n* = 138 (late start)	Placebo vs. rasagiline, rasagiline vs. rasagiline	6 months placebo vs. rasagiline, followed by 6 months rasagiline vs. rasagiline	Rasagiline superior in early start					Over-estimation, difficult interpretation overlapping effect in the open-label-extension, high drop-out rate
Pålhagen et al. 2006 [46]	Placebo: *n* = 76 Selegiline (10 mg): *n* = 81 Later in combination with levodopa (individual dosage)	Placebo vs. selegiline	Monotherapy with placebo or selegiline, later combination therapy with levodopa for up to 7 years	Selegiline superior		No difference between both combination therapies			Methodology not suited to prove an effect on the course of the disease
Olanow et al. 2009 [47]	Rasagiline 1 mg early start: *n* = 300 Rasagiline 1 mg delayed start: *n* = 288 Rasagiline 2 mg early start: *n* = 295 Rasagiline 2 mg delayed start: *n* = 293	Placebo vs. rasagiline, rasagiline vs. rasagiline	36 weeks rasagiline vs. placebo, followed by 72 weeks rasagiline early start vs. delayed start	Rasagiline superior in both concentrations in early start				A little more adverse drug reactions with 2 mg rasagiline	Effect only with 1 mg rasagiline, not for 2 mg. Difference cannot be explained by delayed-start design Delayed-start design also affected by symptomatic effects

**Table 7:** Summary of current meta-analyses and systematic reviews of MAO-B inhibitors in early-stage PD

Publication	Number of studies	MAO-B inhibitor vs. placebo or levodopa	Efficacy vs. placebo	Efficacy vs. levodopa	Safety: fluctuations	Compatibility: dyskinesia, dystonia	Compatibility: non-motor adverse drug reaction	Methodological weakneses/comments
Binde et al. 2018 [48]	27	Rasagiline vs. placebo and as add-on to levodopa Selegiline vs. placebo and as add-on to levodopa Safinamide vs. placebo and as add-on to levodopa	Rasagiline, selegiline, safinamide superior	Not investigated	Not reported	Not more frequently compared to placebo	Not more frequently compared to placebo	In the study relative efficacy values were calculated In remains unclear, which studies were re-analyzed in the study below
Binde et al. 2020 [35]	79	4 dopamine agonists (cabergoline, pramipexole, ropinirole, rotigotine) vs. 3 MAO-B-inhibitors (selegiline, rasagiline, Safinamide (as add-on)) vs. levodopa vs. placebo	Rasagiline, selegiline, safinamide superior	Rasagiline inferior Selegiline inferior	Not reported	Not reported	Not more frequently compared to placebo	In the study relative efficacy values were calculated In remains unclear, which studies were re-analyzed in the study above
Chang et al. 2017 [49]	10	Rasagiline alone or as add-on vs. placebo	Rasagiline superior	Not investigated	Not reported	Not reported	Not reported	
Hauser et al. 2016a [50]	2	Rasagiline vs. placebo	Rasagiline superior	Not investigated	Not investigated	Not investigated	Not investigated	Meta-analysis of the TEMPO and ADAGIO trial
Márquez-Cruz et al. 2016 [34]	5	Levodopa vs. placebo Pramipexole vs. placebo Rasagiline vs. placebo Selegiline vs. placebo	Rasagiline superior Selegiline superior	Levodopa superior	Not reported	Not reported	Less frequently with MAO-B-inhibitors compared to dopamine agonist or levodopa	
Zhuo et al. 2017 [36]	110	10 different drugs incl. selegiline and rasagiline	Rasagiline superior Selegiline superior	Levodopa superior	Not reported	Not reported	Only indirectly by rate of discontinuation which could have been influenced by insufficient efficacy

**Table:** 

Recommendation (new in German guideline, 2023)
The MAO-B-inhibitors selegiline or rasagiline can be used for the monotherapy of early PD, if the relatively mild symptomatic effect appears to be sufficient
Level of consensus: 100%, strong consensus

#### How effective and safe is amantadine compared to placebo or levodopa or dopamine agonists in the monotherapy of early-stage PD?

*Background*: The effectiveness of amantadine on the motor symptoms of PD was described in the 1960s, coincidentally during its use as an antiviral medication in flu-infected PD patients [51].

*Results*: There is no current study investigating amantadine in the monotherapy of PD. Amantadine has an antidyskinetic effect on levodopa-induced dyskinesias. A high-quality, systematic analysis has shown that there is insufficient evidence for the effectiveness and safety of amantadine in the symptomatic treatment of early stages of PD [8]. This was confirmed by a recent review article [9].

**Table:** 

Recommendation (new in German guideline, 2023)
Amantadine should not be used for the monotherapy of early PD
Level of consensus: 96.2%, strong consensus

#### How effective and safe are anticholinergics compared to placebo or levodopa or dopamine agonists in the monotherapy of early-stage PD?

*Background*: The anticholinergics commonly used in the treatment of PD act by selectively blocking muscarinic striatal receptors and modulating dopamine release in the basal ganglia.

*Results*: Studies on efficacy and tolerability of anticholinergics were mostly conducted before the introduction of dopaminergic agents and often do not meet current methodological standards. There are no recent RCTs available. Therefore, there are insufficient data from randomized studies on the efficacy and tolerability of anticholinergics in PD. Short-term and long-term side effects include cognitive impairment and confusion with an increased risk of hallucinations in elderly patients [1]

**Table:** 

Recommendation (new in German guideline, 2023)
Anticholinergics should not be used for the monotherapy of early PD
In absolute exceptional cases, the use of anticholinergics may be considered for otherwise untreatable tremor
Level of consensus: 100%, strong consensus

#### How do age, life circumstances, comorbidities, concomitant medications, and subjective preferences influence the selection of drug classes approved for monotherapy of early-stage PD (levodopa, dopamine agonists, MAO-B-inhibitors, amantadine, anticholinergics)?

*Background*: Age, life circumstances, and comorbidities can have a significant impact on the treatment options for PD.

*Results*: Due to the lack of comparative studies addressing this question, this recommendation is based on expert opinion.

**Table:** 

Recommendation (new in German guideline, 2023)
When selecting different drug classes for initial monotherapy, the varying efficacy in terms of effects, side effects, patient age, comorbidities, and psychosocial profile should be considered
Level of consensus: 100%, strong consensus

#### How does the sex influence the selection of drug classes approved for monotherapy of early-stage PD (levodopa, dopamine agonists, MAO-B inhibitors, amantadine, anticholinergics)?

*Background*: Sex differences might influence the decision for one or the other therapy of PD.

*Results*: A recent review discussed sex-specific differences in movement disorders, which concluded that here are currently no recommendations for sex-specific treatment of PD [52].

**Table:** 

Recommendation (new in German guideline, 2023)
The sex currently has no significance for the selection of PD therapy
Level of consensus: 100%, strong consensus

#### Does the initial selection of drug classes approved for monotherapy of early-stage PD (levodopa, dopamine agonists, MAO-B-inhibitors, amantadine, anticholinergics) influence the long-term course of the disease (e.g., development of fluctuations or dyskinesias)?

*Background*: The treatment for PD is lifelong. The question arises, whether the selection of pharmacological substances for the initial treatment influences the long-term course of the disease and the development of dyskinesias in particular.

*Results*: There are studies comparing the risk of developing fluctuations and dyskinesias under therapy with levodopa with other forms of therapy for PD. In the course of PD, motor fluctuations and dyskinesias inevitably occur. The relationships between levodopa dose, levodopa preparation, disease duration, or patient age remains controversial. There is no therapy with a proven disease-modifying effect [19]. Even though there is no clear proof for a relationship between levodopa dose or use as monotherapy and the development of dyskinesias, clinical experience does suggest a relationship. Therefore, in the consensus meeting, the following recommendations were agreed upon.

**Table:** 

Recommendation (new in German guideline, 2023)
None of the approved anti-Parkinson's medications have proven disease-modifying efficacy
Motor fluctuations and dyskinesias are observed earlier in the disease course after initial monotherapy with levodopa at high doses and pulsatile administration compared to initial monotherapy with MAO-B-inhibitors or dopamine agonists
Preference for dopamine agonists or MAO-B-inhibitors over levodopa should be considered for biologically younger patients
Patients who require levodopa as initial monotherapy should receive it
Reasons for the initial use of levodopa could include:
Severity of symptoms,
Need for a rapid therapeutic effect,
Presence of comorbidities,
Observed or expected side effects of other medication classes (e.g., impulse control disorders with dopamine agonists),
Potentially better individual tolerability
Level of consensus: 100%, strong consensus

### Part III—early combination therapy

#### Should a switch of monotherapy or a combination therapy be considered for PD patients with inadequate efficacy, who have not yet experienced motor complications (fluctuations/dyskinesias)?

*Background*: In some cases, satisfactory symptom control cannot be achieved with the initiation of monotherapy, raising the question if an alternative monotherapy should be chosen or a combination therapy should be started.

*Results*: Regarding the question of switching therapy from one monotherapy to another, no RCTs are available. The best data can be obtained from RCTs where levodopa was allowed as rescue therapy. There is no data to support the notion that switching from one monotherapy to another is beneficial in cases of inadequate efficacy, but there is also no data to prove, that an alternative monotherapy is not an efficient strategy. MAO-B inhibitors are only approved either for monotherapy or as add-on to levodopa to reduce fluctuations in Germany. Adding MAO-B inhibitors to a monotherapy with an agonist, specifically in absence of fluctuations, is off-label in Germany and not supported by published data.

**Table:** 

Recommendation (new in German guideline, 2023)
Based on an initial monotherapy, pharmacological combination therapy should be considered when:
The efficacy of the initial monotherapy at a moderate maintenance dose for dopa-sensitive symptoms is inadequate, or
The dosage necessary for symptom control with a monotherapy cannot be achieved due to therapy-limiting side effects
Starting from an initial monotherapy with a MAO-B-inhibitor, if efficacy is inadequate, a monotherapy or combination therapy with a dopamine agonist or levodopa should be offered
Starting from an initial monotherapy with levodopa, if efficacy is inadequate, a combination therapy with a dopamine agonist should be offered
Starting from an initial monotherapy with a dopamine agonist, if efficacy is inadequate, a combination therapy with levodopa should be offered
Level of consensus: 100%, strong consensus

#### How effective and safe is an early combination of dopamine agonists and levodopa in PD patients who have not yet experienced motor complications (fluctuations/dyskinesias)?

*Background*: In principle, a combination therapy of dopamine agonists and levodopa may be indicated in patients in the early stages of PD. In particular, a combination therapy can be considered useful, if symptoms occur that are in general responsive to dopaminergic medication but do not show sufficient responsiveness to a moderate daily dose of either levodopa or a dopamine agonist.

*Results*: In reference to the STRIDE-PD study [16], priority should be given to a combination therapy at moderate doses rather than a monotherapy at high doses. A combined therapy should be considered particularly, if further increase of the daily dose of levodopa or dopamine agonists is not possible or if a reduction of the daily dose is needed due to intolerable side effects. There are hardly any systematic, randomized, controlled studies on the currently recommended non-ergoline dopamine agonists in combination with levodopa, and thus no sufficient evidence on the efficacy and safety of a combination therapy is available [53, 54]. Currently, the recommendations for combination therapies are therefore expert opinion based primarily on cohort and case observations and pathophysiological considerations.

**Table:** 

Recommendation (new in German guideline, 2023)
PD patients without motor complications (fluctuations/dyskinesias), who exhibit inadequate efficacy on motor symptoms or show limiting side effects under monotherapy with a non-ergoline dopamine agonist at a moderate maintenance dose, should be offered an early combination therapy with levodopa
PD patients without motor complications (fluctuations/dyskinesias), who experience inadequate efficacy on motor symptoms or who present with limiting side effects under monotherapy with levodopa at a moderate maintenance dose (approximately 300 mg/day, adjusted to body weight), should be offered an early combination therapy with a non-ergoline dopamine agonist
The medications should be selected based on efficacy, side effects, patient age, comorbidities, and requirements of the psychosocial profile
A cautious titration of a dopamine agonist in addition to levodopa therapy is especially recommended for patient groups with an increased risk profile for potential side effects
Level of consensus: 100%, strong consensus

#### How effective and safe is levodopa as adjunctive therapy to a MAO-B-inhibitor in PD patients who have not yet experienced motor complications (fluctuations/dyskinesias)?

*Background*: In Germany, there are currently three MAO-B inhibitors available for the treatment of PD: selegiline, rasagiline, and safinamide [55]. The latter has not yet been approved by the authorities in Germany for the early stages of the disease without fluctuations/dyskinesias and can only be used in combination with levodopa. Hence this question focuses on the MAO-B inhibitors selegiline and rasagiline.

*Results*: While the efficacy and safety of rasagiline as monotherapy in the early phase of PD are well established, there are very few systematic, randomized, controlled studies investigating the combination of MAO-B-inhibitors and levodopa in patients without motor fluctuations. There were insufficient data on safinamide to justify its use in combination therapy with levodopa in patients without motor fluctuations.

**Table:** 

Recommendation (new in German guideline, 2023)
Parkinson patients without motor complications (fluctuations/dyskinesias) who experience inadequate improvement in motor symptoms under a monotherapy with the MAO-B-inhibitors rasagiline or selegiline may be offered an early combination therapy with levodopa
In patient groups with an increased risk profile (advanced age, high comorbidity) and a need for additional administration of levodopa to an existing treatment with an MAO-B-inhibitor, the potential for side effects should be considered, and the indication for continuing the MAO-B-inhibitor should be individually and critically evaluated
Level of consensus: 100%, strong consensus

### Part IV – fluctuations and dyskinesia

- Fluctuations

In the first years after diagnosis, most PD patients can achieve a uniform control of motor and some non-motor symptoms with multiple daily doses of dopaminergic medications. However, as the disease progresses, the number of dopaminergic neurons in the substantia nigra continues to decrease and becomes insufficient to release levodopa continuously [56]. Consequently, patients experience a noticeable loss of efficacy toward the end of a dosing interval, i.e., before the next dose of medication is being taken. Initially, this concept of initial fluctuations is implicitly assumed with a dosing frequency of three times a day, corresponding to dosing intervals of approximately 4–5 h.

#### How effective is extended-release levodopa compared to standard-release levodopa in the treatment of PD patients with fluctuations?

*Background*: Levodopa is available in extended-release formulations.

*Results*: Extended-release levodopa formulations achieve maximum plasma concentrations later compared to standard-release levodopa formulations, with approximately similar plasma half-lives, thereby building up effective levodopa levels in the plasma for a slightly longer duration. However, the absorption of extended-release levodopa is more dependent on food intake than the standard formulation [57, 58]. While extended-release levodopa may conceptually reduce motor fluctuations, in clinical practice, there is sometimes an increase in off-time after a switch from immediate-release to extended-release levodopa. This effect may be due to the poorer and less reliable intestinal absorption of extended-release levodopa, as a consequence of interference with food intake. There is no published evidence that extended-release levodopa is superior to standard levodopa during daytime.

**Table:** 

Recommendation (new in German guideline, 2023)
Extended-release formulations of levodopa with dopa decarboxylase inhibitor can in principle be used to treat PD patients with fluctuations. Due to the longer absorption time, these preparations should not be used during waking hours, but only to treat Parkinson's symptoms during nighttime
Level of consensus: 100%, strong consensus

#### How effective is rapidly dissolving levodopa compared to standard-release levodopa in the treatment of PD patients with fluctuations?

*Background*: Rapidly dissolving levodopa has been available for many years and has been developed to potentially enable a faster onset of levodopa action and thus resolve off-states more quickly.

*Results*: There are no controlled studies on the efficacy of rapidly dissolving levodopa used for fluctuations. Therefore, the recommendations are based on expert opinion.

**Table:** 

Recommendation (new in German guideline, 2023)
Rapidly dissolving levodopa can be used to achieve a faster motor improvement upon waking up in the morning compared to conventional formulations
Rapidly dissolving levodopa can be used as on-demand treatment for unexpected off-states during the day
Level of consensus: 100%, strong consensus

#### How effective is inhaled levodopa compared to standard-release levodopa in the treatment of patients with PD experiencing fluctuations?

*Background*: Inhaled levodopa powder is a novel on-demand therapy for treating off-episodes in patients taking oral levodopa in combination with a dopa-decarboxylase inhibitor. Each inhaled application requires a dose of 84 mg, for which patients sequentially load two capsules containing 42 mg each into the inhaler device.

*Results*: A meta-analysis evaluated the results of 4 double-blind randomized studies and 1 open randomized study [59]. Inhaled levodopa resulted in an on-state more frequently than placebo, improved mobility based on the UPDRS motor scale, and led to greater global improvement from the patients’ perspective. Respiratory symptoms (cough, colorless sputum, throat irritation) and dizziness were the most frequently reported adverse effects. There is no direct comparison with other on-demand therapies or with levodopa in standard-release formulation. Real-world data and long-term data on the effectiveness and safety of inhaled levodopa are not yet available.

**Table:** 

Recommendation (new in German guideline, 2023)
Inhaled levodopa can be used as on-demand therapy for daytime off-episodes that occur unexpectedly
Level of consensus: 100%, strong consensus

#### How effective is the additional administration of dopamine agonists compared to placebo in the treatment of PD patients with fluctuations?

*Background*: Additional dopamine agonists are recommended as a treatment of choice for advanced PD patients with motor fluctuations.

*Results*: These recommendations are based on 5 systematic reviews of dopamine agonist therapy in PD with motor fluctuations [37, 39, 60–62] and 1 systematic review of apomorphine infusion [63]. There is a consensus publication on apomorphine treatment [64], a review article [65] and an RCT on sublingual apomorphine [66]. The addition of dopamine agonists to levodopa treatment in advanced PD patients with motor fluctuations results in: 1. Prolonged time in the on-state without troublesome dyskinesias, 2. Reduced time in the off-state, 3. Improved motor symptoms in the on-state (according to UPDRS III), and 4. Improved UPDRS II (ADL) score. These effects are observed with both extended-release (once daily) and standard-release (three times daily) formulations of pramipexole and ropinirole. Impulse control disorders can occur with any dopaminergic treatment, but the risk is greater with dopamine agonist therapy than with levodopa treatment. There is an association between dopamine agonist therapy and increased daytime sleepiness.

Practical advice for dopamine agonist therapy

- Extended-release formulations of pramipexole, ropinirole, and rotigotine primarily offer advantages in terms of medication management and likely improve compliance.
- Caution should be exercised if the patient is older and/or suffers from vascular dysautonomia, has a history of addiction or any impulse control disorder.
- A common observation is that the addition of agonists has a mood-enhancing effect, and pramipexole was the first medication shown in RCTs to have an effect on depression in PD.
- Dopamine agonists should be introduced slowly to reduce side effects. Nausea is usually reversible, while hypotension and peripheral edema often persist. If the patient has difficulty tolerating the initiation with a dopamine agonist, short-term use of domperidone 10 mg three time daily can be considered, taking the associated cardiac risks into account.

**Table:** 

Recommendation (new in German guideline, 2023)
Dopamine agonists can be used to reduce motor fluctuations in patients with advanced PD
Ergoline dopamine agonists (such as bromocriptine, cabergoline, pergolide, lisuride) should no longer be used for the treatment of PD
A dopamine agonist should be titrated up to a clinically effective dose. If side effects prevent this, another agonist or class of drugs can be used
Intermittent subcutaneous apomorphine injections or sublingual apomorphine administration can be used in addition to oral therapy to shorten daily off-periods in patients with severe motor fluctuations
Continuous subcutaneous apomorphine infusion can be used to improve off-periods and dyskinesias in patients with severe motor complications
Level of consensus: 100%, strong consensus

#### How effective is the additional administration of dopamine agonists compared to COMT inhibitors for the treatment of PD patients with fluctuations?

*Background*: There is evidence from controlled studies that in PD patients with motor fluctuations, the addition of a COMT-inhibitor (entacapone, tolcapone, opicapone) or a dopamine agonist (ropinirole, pramipexole, rotigotine, apomorphine) can reduce off-time and improve on-time without troublesome dyskinesia.

*Results*: The effectiveness and risk–benefit profile of dopamine agonists and COMT inhibitors (as well as MAO-B-inhibitors) used as adjunctive therapy in PD patients with fluctuations were evaluated in a Cochrane meta-analysis in 2010 through an indirect comparison [61]. Furthermore, there is one review article [67]. However, there are no direct comparative studies between the adjunctive use of COMT inhibitors and dopamine agonists in PD patients with fluctuations.

**Table:** 

Recommendation (new in German guideline, 2023)
Dopamine agonists or COMT inhibitors can be used for the treatment of motor complications, differing in their individually varying side effect profiles. There is insufficient evidence for a comparative assessment of the efficacy of the two substance classes
Level of consensus: 100%, strong consensus

#### How effective is the additional administration of dopamine agonists compared to MAO-B-inhibitors in the treatment of PD patients with fluctuations?

*Background*: In the advanced stage of PD, a combination therapy is often employed, involving the combination of levodopa with dopamine agonists, COMT inhibitors, MAO-B inhibitors, and other substances.

*Results*: Direct comparative studies between dopamine agonists and MAO-B-inhibitors in advanced stages of PD were not found. There is only one comparative study which included RCTs with patients in different stages of PD [35]. Due to this limited data, no clear recommendation can be made for one of the substance groups.

**Table:** 

Recommendation (new in German guideline, 2023)
A clear recommendation for the preferred use of either class of substances cannot be made
Level of consensus: 100%, strong consensus

#### How effective are intermittent apomorphine administrations compared to standard levodopa in the treatment of PD patients with fluctuations?

*Background*: Apomorphine, the most potent dopamine agonist, can be used to rapidly and reliably terminate off-states that persist despite adjustments of oral therapy.

*Results*: There are reviews [61, 64, 68, 69] and randomized studies [70–72] that showed that subcutaneous apomorphine led to a significant reduction of off-time compared to placebo [68]. Only one study, including 12 participants compared apomorphine injections to oral levodopa. The effect with apomorphine occurred significantly faster than with rapidly dissolving levodopa (after 8.1 vs. 26.8 min); it reached the same level as rapidly dissolving levodopa [73].

**Table:** 

Recommendation (new in German guideline, 2023)
Intermittent subcutaneous apomorphine injections or application of sublingual apomorphine films can be used in addition to oral therapy to reduce the daily off-time in patients with motor fluctuations
Subcutaneous apomorphine injections should only be initiated by experienced physicians and require appropriate monitoring
Level of consensus: 100%, strong consensus

#### How effective is the shortening of levodopa or standard-release dopamine agonist dosing intervals in the treatment of PD patients with fluctuations?

*Background*: In general, levodopa is initially administered 3–4 times per day. Increasing the number of doses could have an effect on motor fluctuations.

*Results*: Levodopa fragmentation is a frequently used strategy [74]. Nevertheless, there is only limited data about the effect of fragmentation of levodopa. In an open-label comparison, there was no difference between a fourth levodopa dose compared to three doses plus a COMT-inhibitor [75]. No randomized controlled trials investigated the effect of more frequent dosing of oral levodopa or dopamine agonists. Insights from clinical studies and clinical experience suggest that at least an adjustment of levodopa medication, usually with more and lower doses, may be effective in reducing off-time.

**Table:** 

Recommendation (new in German guideline, 2023)
An adjustment of levodopa doses (more frequent administrations and shorter dosing intervals, possibly with smaller individual doses) can be made to reduce fluctuations in PD patients experiencing fluctuations
Level of consensus: 100%, strong consensus

#### How effective is the additional administration of MAO-B-inhibitors compared to placebo in the treatment of PD patients with fluctuations, and what differences exist regarding safety and tolerability of the approved MAO-B-inhibitors for the treatment of PD patients with fluctuations?

*Background*: As described above, the MAO-B-inhibitors selegiline and rasagiline have shown significant therapeutic efficacy as monotherapy. Safinamide is only approved for combination therapy with levodopa. All three MAO-B-inhibitors have demonstrated efficacy as add-on to levodopa treatment in patients with motor fluctuations.

*Results*: There are high-quality RCTs for rasagiline and safinamide in advanced patients with fluctuations. These show positive effects of both MAO-B-inhibitors on reducing off-time and increasing on-time without troublesome dyskinesia. A Bayesian network meta-analysis with 31 RCTs and 7142 patients [76] showed a significant improvement in combination therapy of levodopa with selegiline, rasagiline, or safinamide. Consistently, clinical trials and meta-analyses showed that addition of MAO-B-inhibitors led to increased on-time and reduced off-time. The discontinuation rate due to side effects was at the level of placebo with rasagiline, while it was significantly higher with selegiline compared to placebo [77]. The switch from selegiline to rasagiline could mostly be carried out without new side effects [78]. In two meta-analyses, there were no significant differences in the incidence of SAEs between the MAO-B-inhibitors [35, 78]. Despite the lack of comparative data, there is no indication for differences in safety and tolerability of the three available MAO-B-inhibitors.

**Table:** 

Recommendation (new in German guideline, 2023)
If motor fluctuations are not adequately controlled under levodopa therapy, MAO-B-inhibitors can be additionally offered to reduce off-time
The MAO-B-inhibitors are generally well-tolerated. Due to the lack of comparative studies, no recommendation can be made for or against any of the substances within that group
Level of consensus: 100%, strong consensus

#### How effective is the additional administration of COMT inhibitors compared to placebo in the treatment of PD patients with motor fluctuations, and what are the differences in terms of safety and tolerability among the approved COMT inhibitors for the treatment of PD patients with fluctuations?

*Background*: The combination of orally administered levodopa with a dopa decarboxylase inhibitor and a COMT-inhibitor can reduce the extent of plasma level fluctuations of levodopa compared to the combination of levodopa and a decarboxylase inhibitor alone. Clinically, this results in a lower frequency and severity of motor fluctuations, especially off-periods. On the other hand, dyskinesias can occur if the time intervals between intake timepoints are not sufficiently far apart from a pharmacokinetic perspective. Currently, three COMT inhibitors are available for the treatment of fluctuations: entacapone, tolcapone, and opicapone. An important criterion in the selection of the substance beyond effectiveness is safety and tolerability.

*Results*: These recommendations are based on 3 systematic review articles on COMT-inhibitor therapy in PD with motor fluctuations [4, 79, 80], as well as one review on opicapone therapy [81]. In general, COMT inhibitors reduce off-time and fluctuations in patients with levodopa therapy. Only in one RCT, two COMT inhibitors (entacapone and opicapone) are directly compared [7], but systematic reviews [82–84] and network analyses [4] allow for a good indirect comparison. Less data exists for opicapone because it was approved after entacapone or tolcapone. Statements about tolcapone are mainly based on data published prior to the restrictions of use imposed by the European Medical Agency. Overall, opicapone shows the best profile of adverse events, which consists mainly of dyskinesias. Reducing the levodopa dose may be helpful if dyskinesias occur. The safety profile of entacapone is characterized by diarrhea and nausea. Since the substance must be given with every levodopa dose, discontinuation is often unavoidable in these cases. Tolcapone shows the worst safety profile due to the rare but serious cases of hepatotoxicity.

**Table:** 

Recommendation (new in German guideline, 2023)
If motor fluctuations are not adequately controlled under levodopa therapy, an additional COMT-inhibitor can be offered
Opicapone and entacapone are largely equivalent in their efficacy as COMT inhibitors and can be used for the treatment of fluctuations in PD as first-line agents
Tolcapone should only be used as a second-line treatment due to hepatotoxicity concerns, and it should be administered under close safety monitoring (clinical and laboratory)
Level of consensus: 100%, strong consensus

#### How effective is the additional administration of COMT inhibitors compared to MAO-B-inhibitors in the treatment of PD patients with fluctuations?

*Background*: A combination therapy is often used in the advanced stages of PD. This involves combining levodopa with dopamine agonists, COMT inhibitors, MAO-B-inhibitors, and other substances.

*Results*: There are only two RCTs directly comparing the additional administration of a COMT-inhibitor with the administration of an MAO-B-inhibitor. One study compared levodopa with an adjunctive therapy with entacapone or selegiline or both [85]. The other study compared levodopa treatment with an adjunctive treatment with rasagiline, entacapone, or placebo [86]. In both there were no significant differences between the treatment groups. Furthermore, there is one meta-analysis that did not find differences between the two classes of drugs regarding treatment of fluctuations [80]. Thus, a clear recommendation for one class of drugs cannot be made. Additionally, data on safinamide or opicapone are lacking.

**Table:** 

Recommendation (new in German guideline, 2023)
A clear recommendation for the preferential use of one of the two classes of substances cannot be made
Level of consensus: 100%, strong consensus

#### Can a prioritization of the different pharmacological options for treating fluctuations in PD be recommended?

*Background*: As outlined above, various pharmacological treatment options are available for managing fluctuations:

- Fractionation of levodopa doses and possible dose adjustments.
- Additional doses of levodopa preparations with modified formulations (soluble, inhalable, or extended-release levodopa).
- Additional administration of dopamine agonists.
- Additional administration of MAO-B-inhibitors.
- Additional administration of COMT inhibitors.

Evidence-based recommendations exist for each of these individual options. However, in practice, it is necessary to prioritize these options for each individual patient.

*Results*: There are no randomized, controlled studies evaluating the prioritization of the various available options for treating fluctuations in PD patients. Numerous reviews discuss the aforementioned options and provide expert opinions regarding their clinical use [67, 87]. Since there are no controlled studies to answer the question of prioritization, the recommendation relies on the evidence for each individual option and expert opinion.

**Table:** 

Recommendation (new in German guideline, 2023)
A prioritization of the individual therapeutic options for PD patients with fluctuations cannot be made based on studies
The individual sequence of therapy options should take into account the spectrum of efficacy, the side effects profile, and the patient's preference
Level of consensus: 100%, strong consensus

(b) Dyskinesia

*Background*: Dyskinesias are defined as involuntary movements that occur in PD patients after several years of disease duration following treatment with levodopa. They can significantly impair movement and quality of life. The cause of dyskinesias is believed to be a combination of pulsatile dopaminergic stimulation of dopamine receptors over time, associated with increasing degeneration and consequent dysregulation of genes and proteins in downstream neuronal networks, leading to changes in activation patterns [88].

#### How effective is the additional administration of amantadine compared to placebo in the treatment of patients with advanced PD with dyskinesias?

*Results*: The majority of PD patients treated with levodopa develop motor complications in the form of motor fluctuations and dyskinesias over long-term therapy with levodopa. The NMDA-receptor antagonist amantadine has demonstrated consistent effects on reducing dyskinesias in several recent randomized, placebo-controlled studies. A meta-analysis of all studies reinforces the robustness of this effect [89].

**Table:** 

Recommendation (new in German guideline, 2023)
Amantadine should be used to reduce dyskinesias in PD patients with levodopa-induced motor complications, taking into account anticholinergic and hallucinogenic side effects
The use of amantadine requires comprehensive monitoring, especially in geriatric patients, including monitoring for psychiatric side effects, such as hallucinations, renal retention parameters, residual urine, and ECG monitoring, due to potential QT interval prolongation
Level of consensus: 100%, strong consensus

#### How effective is the additional administration of MAO-B-inhibitors compared to placebo in the treatment of patients with advanced PD with dyskinesias?

*Evidence basis*: The effect of safinamide, with a dual mechanism of action as a partial NMDA-receptor antagonist and MAO-B-inhibitor, on the frequency and severity of dyskinesias was examined in 2 studies.

*Results*: The data on the influence of safinamide at doses of 50 mg or 100 mg on the intensity and frequency of dyskinesias is not entirely conclusive. Secondary effects related to levodopa sparing cannot be ruled out with certainty. In a three-arm prospective, randomized, placebo-controlled trial with safinamide over a period of 24 weeks, no statistically significant effect on troublesome dyskinesias was found with either 50 mg or 100 mg of safinamide. However, there was also no increase in dyskinesias despite a significant increase in on-time, which was attributed to an NMDA-receptor antagonistic effect rather than a MAO-B inhibitory effect [90]. In a post hoc analysis investigating the effect of 50 mg and 100 mg of safinamide over a treatment duration of 24 months in PD patients treated with levodopa and experiencing motor complications, a significant reduction in dyskinesia was observed only with 100 mg of safinamide in patients with moderate to severe dyskinesias at baseline [91]. In the group of PD patients without changes in levodopa dose, a reduction of dyskinesias remained significant in a 2-year post hoc analysis at a dose of 100 mg [92].

**Table:** 

Recommendation (new in German guideline, 2023)
Safinamide may be considered for the treatment of moderate to severe dyskinesias
The evidence regarding the efficacy and dose of safinamide on dyskinesias is not conclusive; the partial NMDA-receptor antagonistic effect may be responsible for reducing dyskinesias, and effects due to levodopa sparing cannot be ruled out
Due to lack of evidence, no recommendation can be made for the therapy of dyskinesias with the MAO-B-inhibitors rasagiline or selegiline
Level of consensus: 85.7%, consensus

#### How effective is the additional administration of COMT inhibitors or dopamine agonists compared to placebo in the treatment of patients with advanced PD with dyskinesias?

*Results*: There is insufficient evidence to assess the effectiveness of the class of COMT inhibitors or dopamine agonists in treating dyskinesias.

**Table:** 

Recommendation (new in German guideline, 2023)
Due to insufficient evidence, no recommendation can be made regarding the use of COMT inhibitors or dopamine agonists for the treatment of dyskinesias
Level of consensus: 100%, strong consensus

### Part V—Parkinsonian tremor

Any pathological form of tremor in a Parkinson's patient is classified as Parkinsonian tremor [93]. Rest tremor is a key symptom of PD and presents as the most common motor sign in PD patients, occurring in nearly 90% of patients at some point in the course of the disease [93]. Rest tremor typically appears first in the upper limbs, less commonly in the lower limbs, and then spreads to the other side of the body. Lips, chin, and face can also be affected by rest tremor. One form of rest tremor specific to PD is the pill-rolling tremor, which is characterized by repetitive flexion movements of the thumb and index finger [94].

Additionally, many PD patients also exhibit an action tremor, either in the form of postural and kinetic tremor or in the form of tremor occurring shortly after transitioning from rest to action at rest tremor frequency (rest tremor breakthrough or re-emergent tremor) [93, 95].

#### How effective are dopaminergics (levodopa/dopamine agonists) compared to placebo for treating tremor in PD?

*Background*: Levodopa is considered the most effective oral medication for treating motor symptoms in PD [87].

*Results*: Only a few controlled studies have specifically addressed the efficacy of levodopa in Parkinsonian tremor. Overall, data from three placebo-controlled single-dose studies [96–98] are available on the effect of levodopa on Parkinsonian tremor. These studies, in line with clinical experience, show a good effect of levodopa on Parkinsonian tremor, both for resting and action tremor. From the available data, it cannot be inferred what proportion of patients with Parkinsonian tremor respond poorly to levodopa. Additionally, there are no study data on the effect of higher doses of levodopa on tremor that is poorly responsive to standard doses. Clinical experience shows that tremor refractory to standard doses can respond well to an increase in the daily dose of levodopa or to high single doses of levodopa. Two randomized controlled studies exist on the effect of oral dopamine agonists in the treatment of severe Parkinsonian tremor. Oral dopamine agonists lead to a relevant improvement in Parkinsonian tremor compared to placebo, both in monotherapy and in combination with Levodopa. The conducted studies show that dopamine agonists, like levodopa, improve both resting and action tremor [99, 100]. It is assumed that the effect of dopamine agonists on Parkinsonian tremor is a class effect of dopamine agonists. However, the available data do not allow a definitive conclusion about the effect size of dopamine agonists on Parkinsonian tremor, particularly whether the effect size of dopamine agonists is equal to or weaker than that of levodopa. There is weak evidence that dopamine agonists may have an additional effect on Parkinsonian tremor inadequately treated with levodopa. Therefore, the choice of therapy—levodopa and/or dopamine agonist, in what dose, and in what combination—must be decided individually for each patient.

**Table:** 

Recommendation (new in German guideline, 2023)
Levodopa is used to treat symptoms of PD in all stages. When levodopa is initiated targeting symptoms, such as akinesia and rigidity, Parkinsonian tremor usually improves equivalently. In cases where Parkinsonian tremor is refractory to standard doses of levodopa, increasing the daily dose of levodopa or administering high single doses may be helpful on a case-by-case basis. However, a permanent increase in levodopa dose for poorly treatable tremor should be weighed against the increased risk of motor complications
Dopamine agonists should be used in monotherapy and combination therapy to treat symptoms of PD. It is recommended to initiate treatment targeting symptoms, such as akinesia and rigidity, which usually leads to equivalent improvement in Parkinsonian tremor
Level of consensus: 100%, strong consensus

#### How effective are anticholinergics compared to placebo for treating tremor in PD?

*Background*: Anticholinergics are approved for PD.

*Results*: Although anticholinergics are generally considered effective in treating Parkinsonian tremor, there is no clear evidence for a specific effect of anticholinergics on Parkinsonian tremor [101]. The use of anticholinergics in PD is limited to young, cognitively unimpaired patients with otherwise insufficiently treatable tremor symptoms due to their pronounced side effect profile and the emergence of alternative treatment options [1]. The effect size of anticholinergics on Parkinsonian tremor is likely weaker than that of levodopa. Anticholinergics are contraindicated in older and multimorbid patients. A higher cumulative intake of anticholinergics is associated with an increased risk of cognitive side effects [1, 102, 103].

**Table:** 

Recommendation (new in German guideline, 2023)
The use of anticholinergics in PD patients should only be considered in very rare cases due to their anticholinergic side effects, particularly in cases where tremor is otherwise untreatable. Anticholinergics should not be used in geriatric and/or cognitively impaired patients
Level of consensus: 100%, strong consensus

#### How effective and safe are beta-blockers, primidone, or clozapine compared to placebo for the treatment of tremor in PD?

*Background*: Beta-blockers and primidone have been shown to be effective in treating various tremor syndromes, particularly essential tremor. Primidone and clozapine have been proposed as alternative treatment options for tremor syndromes.

*Results*: In several smaller studies with methodological limitations, it has been shown that beta-blockers like propranolol can lead to an improvement in rest and action tremors in PD [98, 104–106]. In a 2003 Cochrane review on the therapy of Parkinsonian tremor with beta-blockers, however, no sufficient evidence of efficacy was found [107]. Due to the lack of evidence, it is impossible to determine whether beta-blocker therapy is a safe and effective treatment for PD tremor. Overall, the reporting of adverse effects was poorly documented. However, the high frequency of a significant decrease in heart rate observed in one trial raises concerns about the safety of beta-blocker therapy.

The data on primidone for the treatment of Parkinsonian tremor is inadequate. Clinical experience shows no evidence of a positive effect.

There are two randomized controlled trials examining the effect of clozapine on Parkinsonian tremor [108, 109]. Clozapine led to a significantly greater reduction in tremor compared to placebo for up to 5 h after administration. Clozapine leads to a significant improvement in Parkinsonian rest and postural tremor compared to placebo. Open-label studies have also shown efficacy in Parkinsonian tremor refractory to other medications such as anticholinergics. A limiting factor for the use of clozapine is the risk of sometimes severe side effects, including agranulocytosis [110].

**Table:** 

Recommendation (new in German guideline, 2023)
Beta-blockers may be considered for the treatment of PD tremor
Level of consensus: 96%, strong consensus

**Table:** 

Recommendation (new in German guideline, 2023)
Primidone should be avoided in the treatment of Parkinsonian tremor due to inadequate data
Level of consensus: 93.3%, strong consensus

**Table:** 

Recommendation (new in German guideline, 2023)
The use of clozapine for the treatment of Parkinsonian tremor can be considered (off-label) taking into account the spectrum of side effects, when other medications are not sufficiently effective or contraindicated, when surgical Parkinson's therapy is not desired or contraindicated, and when adequate monitoring of side effects is ensured
Level of consensus: 96%, strong consensus

#### Under what circumstances is a specific medication or invasive therapy necessary for the treatment of tremor in PD?

*Background*: Following the initial diagnosis, treatment should first focus on target symptoms, such as akinesia and rigidity [100, 101, 111–113]. Generally, this also improves the Parkinsonian tremor accordingly.

*Results*: Levodopa and dopamine agonists represent the most effective medication-based therapy for the symptoms of Parkinson's disease (PD), including Parkinsonian tremor, and are used at all stages. When stage-appropriate medication treatment for PD focuses on target symptoms, such as akinesia and rigidity, the Parkinsonian tremor generally improves equivalently. In addition to medication therapy, effective invasive procedures are also available for the treatment of Parkinsonian tremor, which are addressed in another article in this collection.

**Table:** 

Recommendation (new in German guideline, 2023)
The initial treatment of Parkinson's patients with tremor follows the general therapeutic principles. The choice of initial medication depends on clinical factors, such as age, comorbidities, and the severity of motor symptoms. Once medications have been titrated up according to the requirements for the target symptoms of akinesia and rigidity, invasive therapies including deep brain stimulation (DBS) may be considered to treat any residual tremor
Level of consensus: 100%, strong consensus

### Part VI—special treatment situations

- Perioperative management

#### How should pharmacotherapy for PD be safely and effectively adjusted perioperatively?

*Background*: In literature, there is consensus that the perioperative risk is generally increased in PD patients [114–116]. Reasons cited in this context include dysphagia, impaired pulmonary ventilation due to compromised respiratory muscles [117], a higher risk of falls, and urinary tract infections [118]. Perioperative adjustment of dopaminergic medication becomes necessary when the usual dopaminergic medication needs to be interrupted for more than a few hours during surgery, such as during abdominal surgical procedures that require a longer postoperative fasting period for patients [114], in cases of peri-/postoperative intestinal absorption disorders, or when nutrition via nasogastric tube is required following surgery, for example, in cases of prolonged ventilation or post-ventilation [116].

*Results*: Since there are currently no systematic, randomized, controlled studies, and thus no validated evidence regarding the optimal perioperative adjustment of pharmacotherapy for PD, the recommendations for therapy are based on available cohort and case observations and pathophysiological considerations, in the form of expert opinion.

**Table:** 

Recommendation (new in German guideline, 2023)
For safe and effective switching of pharmacotherapy in PD patients, the following medications can be considered:
1. Levodopa: if oral administration of medication via a tube is possible: calculation of levodopa equivalent dose (LEDD) and administration of soluble levodopa via a gastric feeding tube
2. Rotigotine transdermal patch:
3. For patients who have not received dopamine agonists before, a starting dose of 2–4 mg/24 h rotigotine may be recommended, with gradual titration over several days based on tolerability
4. For patients previously receiving pramipexole, switching to rotigotine at a ratio of 1:4 may be recommended
5. Ropinirole can be switched to rotigotine at a ratio of 1–1.5:1
6. Caution: the patch must be removed before MRI examinations and cardioversions due to its aluminum layer
7. Continuous subcutaneous apomorphine infusion using a pump: target daily doses between 20 and 40 mg/day can be aimed for, if needed under protection against side effects with domperidone
8. Amantadine: intravenous administration of 200 mg amantadine up to three times daily is possible. Alternatively, 500 mg amantadine can be administered once daily for reasons of practicability and for non-geriatric patients that are not prone to delirious syndromes. Caution: renal insufficiency, hallucinations, and delirious syndromes should be monitored
9. General recommendations:
10. Whenever possible, a timely return to the patient's original Parkinson's medication regimen should be aimed for
11. Antidopaminergic antiemetics and neuroleptics with anti-dopaminergic properties should not be used
12. Supportive measures, such as early mobilization, rehydration, and dysphagia evaluation, should be carried out simultaneously to facilitate a rapid return to oral pharmacotherapy
Level of consensus: 95.2%, strong consensus

(b) Freezing of gait

#### How effective are dopaminergic and non-dopaminergic substances compared to placebo for the treatment of freezing of gait in PD?

*Background*: Freezing of Gait (FoG) is a paroxysmal inability of PD patients to initiate or continue walking movements, usually lasting several seconds. Subjectively, patients experience FoG as if their “feet are stuck to the ground” while the upper body continues to follow a propulsion trajectory, resulting in an increased risk of falls [119–121]. Limitation of the current literature is the oversimplified assessment of FoG (e.g., assessment of one single item of the UPDRS) and the lack of FoG-subtype classification.

*Results*: Level-I studies demonstrate the effectiveness of levodopa [17] and the MAO-B-inhibitors selegiline [122] and rasagiline [86] compared to placebo in the treatment of FoG. Levodopa is superior to dopamine agonists, such as pramipexole [123] and ropinirole [124], in level-I studies for treating FoG, although the increased risk of motor fluctuations with levodopa should be considered. All dopaminergic substances are approved for use in PD therapy in Germany. The evidence regarding the use of not-dopaminergic medication such as nicotine bitartrate or rivastigmine for treating FoG is not conclusive in Level-I studies, however, both substances of improved aspects of the PD gait disorder [125, 126]. Methylphenidate has been studied in three level-I studies with heterogeneous results [127–131], but the largest multicenter study showed positive effects on FoG [132]. The evidence for amantadine is heterogeneous, but in extended-release formulation, positive effects on FoG were observed in two phase 3 studies [133]. Adenosine receptor blockers such as caffeine or istradefylline have been studied in small, prospective studies with a positive effect on FoG [134, 135].

**Table:** 

Recommendation (new in German guideline, 2023)
Levodopa is recommended as a symptomatic therapy for freezing of gait (FoG)
It is recommended to prefer levodopa over dopamine agonists, such as pramipexole and ropinirole, for the treatment of FoG at the cost of increased risk of dyskinesias
MAO-B-inhibitors, such as selegiline and rasagiline, are recommended for symptomatic treatment of FoG in early PD
The use of cholinergic stimulants such as nicotine or rivastigmine for treating FoG may be considered
The use of the noradrenergic substance methylphenidate may be considered for treating FoG
Amantadine in the oral extended-release form may be considered for treating FoG
Adenosine receptor antagonists such as caffeine may be considered for treating FoG
Level of consensus: 91.7%, consensus

(c) Pregnancy and breastfeeding

*Background*: Pregnancy and breastfeeding are special situations where pharmacotherapy must be managed with due care to avoid harming the unborn or newborn baby.

#### How must pharmacotherapy for PD during pregnancy be carried out safely and effectively?

*Results*: No randomized controlled trial was found that investigated the treatment of PD during pregnancy. The literature search yielded 9 review articles that were essentially based on case reports and case series, as well as a pharmacovigilance study on dopaminergic medication during pregnancy for restless legs syndrome. In the 2013 pharmacovigilance study, which included 59 pregnancies under treatment with levodopa, pramipexole, ropinirole, or rotigotine, no evidence of an increased risk of malformations was found with these therapies [136]. In a case series from Turkey, complications were reported in 4 out of 15 pregnancies under medication for PD. These treatments involved rasagiline plus pramipexole, pramipexole as monotherapy, piribedil plus levodopa/benserazide, and rasagiline plus levodopa/benserazide [137]. The available data on pregnancy in patients with PD were summarized in a recent review article. According to this, there are no data indicating a higher complication rate for pregnancy in patients with PD. Regarding medication, most data were available for Levodopa, and no increased rates of malformations were reported with Levodopa [138]. It should be noted that according to the individual product information the dopa decarboxylase inhibitor benserazide is contraindicated in pregnancy. Amantadine is teratogenic in both animals and humans.

**Table:** 

Recommendation (new in German guideline, 2023)
If dopaminergic medication is necessary during pregnancy, levodopa in combination with carbidopa should be considered
Due to insufficient data, dopamine agonists and MAO-B-inhibitors should be avoided during pregnancy
Amantadine and the decarboxylase inhibitor benserazide are contraindicated during pregnancy
Level of consensus: 92.3%, consensus

#### How should pharmacotherapy for PD be carried out safely and effectively during breastfeeding?

*Results*: There are no controlled studies or case reports on pharmacological therapy for PD during breastfeeding. The available data on pharmacological therapy for PD during breastfeeding do not allow for recommendations regarding medication therapy during breastfeeding. For pragmatic reasons, pharmacological treatment for PD should be avoided during breastfeeding or breastfeeding should be discontinued.

**Table:** 

Recommendation (new in German guideline, 2023)
Due to the insufficient data available, breastfeeding should be avoided during pharmacological treatment for PD
Level of consensus: 100%, strong consensus

## Discussion

In this new German guidelines for PD, pharmacotherapy of motor symptoms is addressed in five chapters, along with an additional chapter covering special treatment situations.

This article first presents the currently available medications for PD. These guidelines are the first German guidelines to address questions regarding different formulations of levodopa. Furthermore, they cover the prioritization of different drugs within each class. For dopamine agonists, there is no evidence that ergoline dopamine agonists are beneficial when other approved drugs are ineffective. Due to potentially severe side effects, ergoline dopamine agonists are no longer recommended and have thus been removed from the recommendations. With regard to COMT inhibitors, opicapone and entacapone are similarly effective. Tolcapone should only be used as second-line, because of its safety profile. Even though, the data base is largest for rasagiline, there are no data that suggest that one of the MAO-B-inhibitors rasagiline or selegiline should be prioritized over the other and thus no recommendation was made. Since the last guidelines from 2014, safinamide, another MAO-B-inhibitor, has been newly approved. This drug with an additional glutamate-modulating effect is not approved for monotherapy of PD, but can be used adjunctive to levodopa. The only NMDA-receptor antagonist recommended for special treatment situations in PD, such as levodopa-induced dyskinesia, is amantadine. There is no evidence of a beneficial effect of amantadine in early PD stages, so it should not be used at that stage. While the previous version of the guidelines from 2014 did not recommend anticholinergics as a first choice, the current guidelines generally advise against their use, except for cases of otherwise uncontrollable tremor with special regard on potential side effects. The current guidelines update dosage recommendations and remove those for ergoline dopamine agonists. They include safinamide in the MAO-B inhibitor recommendations. Two new papers addressing equivalent doses were used to update the equivalent dosage table, which now includes levodopa-entacapone-carbidopa intestinal gel, opicapone, and safinamide.

In early PD, treatment should be initiated with MAO B-inhibitors, dopamine agonist, or levodopa considering the individual situation of each patient. In biologically younger patients, MAO B-inhibitors or dopamine agonists should be considered. Patients who require levodopa, should receive it. There are still no systematic studies on the best starting point for PD medication, so the recommendations for initiation of therapy remained unchanged and multifactorial. The recommendations on the effectiveness of different drug classes have been updated with information from recently published studies. For dopamine agonists and MAO-B inhibitors, updated tables summarizing recent studies, reviews, and meta-analyses have been included in the current guidelines. For the first time, the influence of the patient's sex on therapy was addressed, but no strong evidence was found to suggest that sex needs to be considered when selecting the appropriate therapy. Additionally, the potential effects of medications on the development of dyskinesias were discussed. The relationship between the development of dyskinesias and factors, such as levodopa dose, disease duration, or patient age, remains controversial. There is still no approved medication with a proven disease-modifying effect.

Moreover, the current recommendations address whether monotherapy should be changed if the first choice is ineffective or limited by side effects, and discuss combination therapies for patients who have not yet experienced motor complications.

The pharmacotherapeutic options for managing fluctuations and dyskinesia are discussed in relation to various drugs and drug classes. The use of different levodopa formulations (e.g., standard-release, extended-release, rapidly dissolving, inhaled levodopa) is specifically addressed. Additionally, therapeutic options adjunct to levodopa (i.e., dopamine agonists, COMT inhibitors, MAO-B inhibitors) are compared. While dopamine agonists, COMT inhibitors, and MAO-B inhibitors are all effective in reducing motor complications, a clear recommendation for one drug class over another cannot be made due to a lack of comparative data. Moreover, the use of apomorphine injections was discussed, emphasizing it should be initiated only by experienced physicians. Fragmentation of levodopa intake is also a potential strategy to reduce fluctuations. For dyskinesia, data show that amantadine is effective, while there is still no evidence that MAO-B inhibitors, COMT inhibitors, or dopamine agonists are effective for this indication. Therefore, these latter drug classes are not recommended for reducing dyskinesia.

With regard to the treatment of PD tremor, levodopa remains the best treatment for all symptoms of PD, including tremor. Unlike previous guidelines, which considered the use of anticholinergics as a “can be used” recommendation for tremor, the present guidelines state that anticholinergics should be considered only in very rare cases of tremor. The recommendation for beta-blockers for PD tremor remains unchanged. They can be considered. While the previous guideline did not recommend primidone for PD tremor due to a lack of data, the current guideline explicitly advises against its use for the same reason. Additionally, the new guideline includes a “can be considered” recommendation for the off-label use of clozapine for PD tremor.

Moreover, the new guideline addresses special treatment situations, such as perioperative management, freezing of gait, pregnancy, and breastfeeding.

This article covers treatment for motor symptoms of PD and special treatment situations. Device-assisted therapy is covered in a different article in this collection. This guidance aims to represent the current best practices as of early 2024 and will be periodically updated.

This guideline was systematically developed aids for doctors to make decisions in specific situations. It is based on current scientific knowledge and proven practices, ensuring more safety in medicine. The recommendations are tailored to the contexts of Germany, Austria, and Switzerland and may not be fully applicable outside these countries. Given the brevity of this summary, we highly recommend referring to the complete, original guideline in German for comprehensive information. This guideline is not legally binding for doctors; the medical assessment of each individual case is always decisive. Therefore, deviations from the guidelines do not create liability, nor does adherence to them absolve liability. Members of this guideline group compiled and published this guideline with the greatest care but cannot assume legal responsibility for their accuracy. Particularly for dosage information regarding drugs or specific substances, the manufacturer's details in the product information must always be considered, along with the individual benefit-risk ratio for the patient and their conditions as assessed by the treating doctor.

## Data Availability

In the present article, a data availability statement is not applicable. No primary data were used. All recommendations are based on the German guideline and the references therein, which were also included in this article.
